# Eczema in early childhood increases the risk of allergic multimorbidity

**DOI:** 10.1002/clt2.12384

**Published:** 2024-09-01

**Authors:** L. A. Miltner, J. M. Vonk, J. L. van der Velde, A. B. Sprikkelman

**Affiliations:** ^1^ Department of Dermatology University of Groningen University Medical Center Groningen Groningen The Netherlands; ^2^ Department of Epidemiology University of Groningen University Medical Center Groningen Groningen The Netherlands; ^3^ Groningen Research Institute for Asthma and COPD (GRIAC) University of Groningen, University Medical Center Groningen Groningen The Netherlands; ^4^ Department of General Practice and Elderly Care Medicine University Medical Center Groningen University of Groningen Groningen The Netherlands; ^5^ Department of Pediatric Pulmonology and Pediatric Allergy University of Groningen University Medical Center Groningen Groningen The Netherlands

**Keywords:** allergic disease trajectory, allergic multimorbidity, eczema, food allergy

## Abstract

**Background:**

Eczema in early childhood is associated with the development of subsequent allergic diseases, including food allergy (FA), asthma and hay fever. However, eczema has a heterogenous presentation regarding onset age and persistence, which may lead to different allergic outcomes during childhood/adolescence. Recently, sub‐phenotypes of eczema have been suggested as predictors of allergic multimorbidity. Thus, we aimed to identify associations of eczema phenotypes with FA, asthma and hay fever during childhood/adolescence. Additionally, we described the trajectories of eczema, asthma and hay fever stratified by FA presence.

**Methods:**

TRACKER (Trajectories of Allergy in Children in Real Life Databases) is a population‐based cohort study of 6852 children/adolescents from the Lifelines cohort. We investigated the associations of seven eczema phenotypes, based on onset age and persistence, with FA, asthma and hay fever using logistic regression, adjusted for appropriate covariates. Disease trajectories were determined by calculating prevalence at different ages.

**Results:**

Participants who suffered from eczema throughout childhood showed higher risks of developing FA, hay fever and asthma. “Very early onset—persistent” eczema showed the strongest associations with FA, asthma and hay fever. The prevalence of eczema, asthma and hay fever at all ages was significantly higher in participants with FA, compared to those without.

**Conclusion:**

One of the largest cohort studies on this topic to date shows that (very) early onset and persistent eczema increases the risk of allergic multimorbidity. Identification of infants at risk for developing (very) early onset eczema is of utmost importance to prevent allergic multimorbidity.

## 1 INTRODUCTION

Trajectories of allergic diseases have often been reported to follow the paradigm of the atopic march, describing the idea of sequential development of allergic diseases, namely atopic dermatitis (AD), food allergy (FA), allergic asthma (AA) and allergic rhinitis (AR), in early life.^1^ AD, also known as eczema, is a chronic, recurrent skin disease, characterized by chronic skin barrier impairment, inflammation of the skin, eczematous lesions and pruritus.^2^ AD develops during the first year of life in about 60% of affected children.^3^ Similarly, primary FA, characterized by IgE‐mediated responses to foods,^4^ generally develops during infancy and early childhood.^5^ AA and AR typically show a later onset and are thus viewed as the later manifestations in the trajectory of allergic diseases.^1^, ^5^

Dysfunction of the skin barrier, a hallmark of AD, plays an important role in the etiology of comorbid allergic diseases. Systemic sensitization against food allergens and inhalant allergens at a young age is thought to be promoted by skin barrier impairment (epicutaneous sensitization), increasing the likelihood for later development of FA, AA and AR.^6^

Recently, studies focusing on childhood AD have established the importance of differentiating between phenotypic subclasses of AD. Typically, these disease phenotypes are being stratified according to age of onset, persistence and severity of AD.^7^, ^8^, ^9^, ^10^, ^11^ Differences in underlying pathophysiological pathways which might cause this heterogeneity of AD are yet to be described.^8^ However, pinpointing the phenotype at the highest risk for developing allergic multimorbidity and identifying phenotype‐specific risk factors is needed for developing personalized prevention and treatment strategies for children with AD.

Thus, we hypothesize that children with early onset and persistent eczema are at the highest risk for sensitization to food and/or inhalant allergens and the subsequent development of allergic multimorbidity. The current TRACKER (Trajectories of Allergy in Children in Real Life Databases) study aims to identify the associations of eczema phenotypes with the presence of FA, asthma and hay fever. In addition, we aimed to describe the trajectories of eczema, asthma and hay fever, differentiating between children with and without FA.

## 2 MATERIAL AND METHODS

### 2.1 Study design and population

Lifelines is a multi‐disciplinary prospective population‐based cohort study examining the health and health‐related behaviors of 167,729 persons living in the North of the Netherlands. It employs a broad range of investigative procedures in assessing the biomedical, socio‐demographic, behavioral, physical and psychological factors which contribute to the health and disease of the general population, with a special focus on multi‐morbidity and complex genetics. Baseline examinations in individuals aged 6 months to 93 years took place between 2006 and 2013 with follow‐up visits every 5 years and follow‐up questionnaires in between.^12^ The Lifelines cohort study was approved by the Medical Ethics Committee of the University Medical Center Groningen (UMCG), Groningen, The Netherlands (2007/152). All subjects gave written informed consent.

For the TRACKER study, subjects between 4 and 18 years of age at baseline, who had completed the questionnaire about allergic diseases and a follow‐up questionnaire concerning food allergies (performed between 2014 and 2017), were included.

### 2.2 Questionnaires

For children 13 years or younger at the time of baseline or follow‐up, parents/guardians completed the questionnaires. For children over 13 years old, both parents/guardians and the child completed questionnaires. At baseline, different versions of the questionnaire covered predefined age brackets (Supplementary Table S1). The version that participants received was dependent on the participant age at the time of the questionnaire (ageQ). The presence of eczema, asthma, and hay fever within each age bracket was retrospectively reported at baseline. Presence of FA was reported at follow‐up.

### 2.3 Definitions

#### 2.3.1 Eczema presence and eczema phenotypes

Atopic dermatitis was operationalized as self‐/guardian‐reported eczema. Within each age bracket, eczema presence was determined using three parameters: presence, medication and treatment by a doctor. The questions for the assessment of the parameters are listed in the supplement. Eczema presence was confirmed if presence was reported or if presence was denied, but either medication or treatment were reported. Eczema was absent if all three parameters were reported negatively. Ever eczema was defined as eczema presence in at least one of the age brackets.

Seven longitudinal phenotypes were defined, based on the presence, age of onset and persistence of eczema (Table 1). These criteria have been utilized previously to differentiate between phenotypes of eczema.^7^ Age of eczema onset was defined as “Very early” if the first presence was reported before the age of 6 months, “Early” between 6 months and 4 years and “Late” after the age of 4 years (Supplementary Figure S1). Persistence/remission of eczema was defined by assessing eczema presence in the most recent age bracket available. When persistence/remission could not be determined due to incomplete data, subjects were classified into phenotypes with “no information on persistence.” Very young children (age <4 years at baseline) provided no information about persistence/remission and were excluded.

**TABLE 1:** Definitions of the eczema phenotypes.

Eczema phenotype	Definition	*N* (%)
Never eczema	No presence of eczema was reported	4877 (71.2)
Very early onset—remitting	Onset between 0—6 m, no eczema presence in most recent age bracket	531 (7.6)
Very early onset—persistent	Onset between 0—6 m, eczema presence in most recent age bracket	269 (3.9)
Very early onset—no info on persistence	Onset between 0—6 m, no data after 6 m	23 (0.3)
Early onset—remitting	Onset between 6 m–4 y, no eczema presence in most recent age bracket	354 (5.2)
Early onset—persistent	Onset between 6 m–4 y, eczema presence in most recent age bracket	206 (3.0)
Early onset—no info on persistence	Onset between 6 m–4 y, no data after 4 years	22 (0.3)
Late onset	Onset after 4 y.	356 (5.2)

Abbreviations: m, months; y, years.

#### 2.3.2 Food allergy presence

Presence of FA was assessed at follow‐up and participants were assigned to one of three groups, previously reported by Westerlaken‐van Ginkel et al. in adults.^13^ The groups represented those who did not report FA (*noFA*), those likely to have a FA (*likelyFA*) and those who could not be assigned to either of those groups, termed indeterminate (*indeterminateFA*). In short, participants were classified as *likelyFA* if they stated at least one food and at least one symptom consistent with immediate allergic reactions to food as well as other characteristics of FA consistent with immediate allergic reactions to food. These included the qualification of the person diagnosing FA and the time of symptom onset after ingestion.

#### 2.3.3 Hay fever

The definition of hay fever presence was identical to the definition of ever eczema presence described above. The presence of hay fever was assessed for each age bracket and over the entire study period.

#### 2.3.4 Asthma

Presence of asthma was based on the medication and treatment parameters. If either question for medication or treatment by a doctor had been answered with “yes”, presence of asthma was confirmed. Again, presence within the age brackets and ever asthma was assessed.

Additional details on the disease definitions can be found in the supplementary.

#### 2.3.5 Age of onset

Age of onset was defined as the mid‐point of the age bracket in which the presence was first reported. For example, if eczema presence was first reported in the period 4–7 years, age of eczema onset was taken as 5.5 years. In cases of age brackets where the end was defined as ageQ, the age of onset represented the mid‐point of the age at the start of the period and ageQ (4 years—ageQ, age 5 at time of questionnaire, age of onset: 4.5 years).

#### 2.3.6 Covariates

Multiple potentially confounding variables were assessed to correct for biases in the statistical analysis:

- Basic characteristics: age at baseline, age at follow‐up, sex
- Information about the pre‐natal phase: smoking during pregnancy and passive smoking during pregnancy
- Information about the post‐natal phase: the location of residency in the first 6 months (*farm*, *rural village*, *small town/large village*, *suburb or large city*, *inner city*, *do not remember*, *unknown*), exposure to a furry/hairy pet in the first 6 months, whether the infant was breast‐fed and the duration of breastfeeding (*not breast‐fed*, *up to* 3 months, *more than* *3 months*, *breastfed but do not remember duration*, *do not know if breast‐fed*)
- Parental characteristics: asthma presence among parents, their income level/month (*no information on income*, < *1500 €*, *1500—3000 €*, > *3000 €*) and their education level (*low*, *medium*, *high*, *other/unknown*)

### 2.4 Statistical analysis

Descriptive analysis of the baseline characteristics, disease phenotypes and covariates of the study population was performed, stratifying by the presence of FA. Continuous and categorial variables were tested using independent *t*‐test and Chi^2^‐test, respectively. To investigate the associations of ever eczema and eczema phenotypes with FA presence, multinomial logistic regression analysis with and without adjustment for all covariates (excluding age at baseline) was conducted. Association of ever eczema and eczema phenotypes with presence of asthma and hay fever were analyzed using logistic regression analysis with and without adjustments for all covariates (excluding age at follow‐up). The trajectories of the different allergic diseases in the study population, stratified by FA presence, were based on the prevalence of eczema, asthma and hay fever within each age bracket. Differences in prevalence of each allergic disease between the FA groups were tested using a Chi^2^‐test with post hoc group‐wise comparison within each age bracket.

Data management tasks and statistical analysis were carried out using R Studio (2022.02.0443, based on R version 4.1.2, RStudio Team (2022), RStudio: Integrated Development Environment for R. RStudio, PBC, Boston, MA URL http://www.rstudio.com/). Relevant packages are listed in the supplement. The significance level for all tests conducted was a *p*‐value of 0.05.

## 3 RESULTS

### 3.1 Study population

**FIGURE 1:**
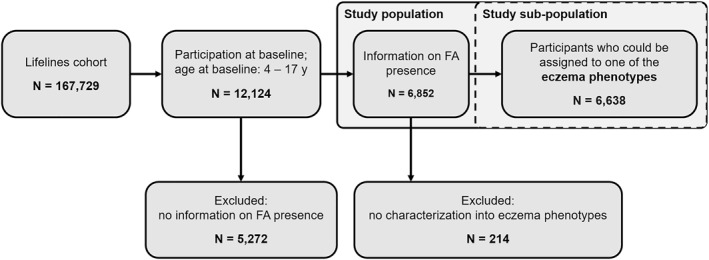
Flow diagram of participant inclusion for the TRACKER study. FA, food allergy; y, years.

Data on eczema and FA were available for 6852 participants, who had a mean age of 9.9 years (SD 3.4) at baseline, with 47.2% of them being male. Of these, 6638 could be assigned to one of the eczema phenotypes (Figure 1). Among the included 6852 children, 27.0% reported eczema, 9.9% asthma and 8.2% hay fever. The distribution of eczema phenotypes and covariates is presented in Table 2 (Supplementary Figure S2A). “Very early onset—remitting” eczema showed the highest prevalence, followed by “Early onset—remitting” and “Late onset”. In total, 6.2% of participants were classified as having *likelyFA* and 1.2% as having *indeterminateFA* (Supplementary Figure S2B). The average onset age for eczema, asthma and hay fever was 2.7 (SD 2.7) years, 3.4 (SD 3.9) years and 7.4 (SD 4.4) years, respectively.

**TABLE 2:** Baseline characteristics and covariates of the TRACKER study population.

Characteristic	Cohort [*N* (%)] (*N* = 6852)	Prevalence of FA (%)				
		No FA (*N* = 6345; 92.6%)	Indeterminate FA (*N* = 84; 1.2%)	*p*‐value no FA—Ind. FA	Likely FA (*N* = 423; 6.2%)	*p*‐value No FA—L. FA
Age—at baseline^a^	9.9 (3.4)	9.9 (3.4)	10.6 (3.5)	0.108	10.3 (3.5)	**0.036**
Age—at follow‐up^a^	12.9 (3.7)	12.8 (3.7)	13.7 (3.8)	0.079	13.2 (3.77)	0.056
Sex—male	3236 (47.2)	47.5	31.0	**0.004**	46.3	0.678
Ever eczema	1853 (27.0)	25.0	42.9	**< 0.001**	55.3	**< 0.001**
Ever asthma	677 (9.9)	9.0	16.7	**0.026**	21.5	**< 0.001**
Ever hay fever	560 (8.2)	6.6	13.1	**0.030**	31.4	**< 0.001**
Eczema phenotypes				**< 0.001**		**< 0.001**
Never eczema^b^	4877 (71.2)	73.2	53.6		43.7	
Very early onset—remitting	531 (7.6)	7.2	*N* < 10		15.1	
Very early onset—persistent	269 (3.9)	3.0	*N* < 10		17.3	
Very early onset—no info	23 (0.3)	0.3	0.0		0.5	
Early onset—remitting	354 (5.2)	5.0	*N* < 10		7.8	
Early onset—persistent	206 (3.0)	2.7	*N* < 10		6.4	
Early onset—no info	22 (0.3)	0.3	0.0		0.2	
Late onset	356 (5.2)	5.2	*N* < 10		5.7	
NA	214 (3.1)	3.1	*N* < 10		3.3	
Residency location first 6 months				0.907		0.052
Other^b^	121 (1.8)	1.8	*N* < 10		1.0	
Farm	344 (5.0)	5.2	*N* < 10		1.9	
Rural village	3034 (44.3)	44.3	40.5		44.2	
Small town/large village	2197 (32.1)	31.8	35.7		35.2	
Suburb or large city	809 (11.8)	11.7	11.9		13.0	
Inner city	260 (3.8)	3.8	*N* < 10		3.8	
NA	87 (1.3)	1.3	0.0		1.0	
Exposure to furry/hairy pet	3665 (53.5)	53.8	58.3	0.744	48.0	**0.042**
Breastfed	5580 (81.4)	81.5	78.6	0.710	80.9	0.830
Duration of breastfeeding				0.624		0.112
Not breast‐fed^b^	1163 (17.0)	16.9	20.2		17.5	
Up to 3 months	2354 (34.4)	34.7	27.4		30.3	
More than 3 months	3181 (46.4)	46.2	50.0		49.2	
Breastfed, no information on duration	45 (0.7)	0.6	*N* < 10		1.4	
NA	109 (1.6)	1.6	*N* < 10		1.7	
Smoking during pregnancy	353 (7.4)	7.6	*N* < 10	0.564	6.4	0.651
Passive smoking during pregnancy	1245 (18.2)	18.4	*N* < 10	**0.045**	17.3	0.675
Asthma presence among parents	1046 (15.3)	14.8	17.9	0.530	22.2	**< 0.001**
Parents income				0.633		0.052
<1500 euro^b^	348 (5.1)	5.2	*N* < 10		3.3	
1500–3000 euro	2682 (39.1)	38.9	39.3		43.0	
>3000 euro	3172 (46.3)	46.3	41.7		47.0	
NA	625 (9.1)	9.5	11.9		6.6	
Parents education				0.560		0.328
Low^b^	121 (1.8)	1.8	0.0		2.4	
Middle	3038 (44.3)	44.6	41.7		40.7	
High	3678 (53.7)	53.4	58.3		57.0	
NA	15 (0.3)	0.3	0.0		0.0	

Abbreviations: FA, food allergy; *N*, number; NA, not available.

^a^ Age variables as mean (standard deviation (SD)).

^b^ Reference attribute for the covariate, *N* < 10: Due to privacy protection, categories with fewer than 10 subjects are displayed as *N* < 10; Significant *p*‐values are depicted bold.

### 3.2 Association between eczema phenotypes and the presence of FA

**FIGURE 2:**
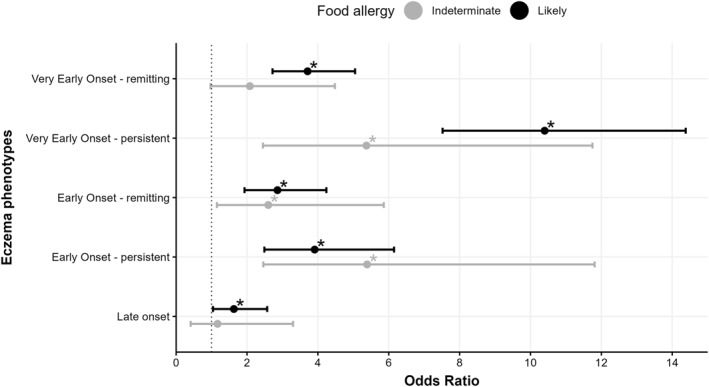
Association between eczema phenotypes and food allergy presence. Adjusted Odds Ratio (with 95% CI) from the multinomial logistic regression analysis are depicted, adjusted for age at follow‐up, sex, smoking during pregnancy, passive smoking during pregnancy, location of residency in the first 6 months, exposure to a furry/hairy pet in the first 6 months, breast feeding, duration of breastfeeding, asthma presence among the parents, parent's income level, parent's education level. Eczema phenotypes were compared to the “Never eczema” reference group. *N* = 6,638, * *p*‐value <0.05.

Participants who suffered from eczema at any point in childhood showed 3.7 times higher odds of developing *likelyFA* and a 2.4 times higher risk for *indeterminateFA* (Supplementary Table S2). The risk of developing *likelyFA* was significantly higher across all eczema phenotypes when compared to “Never eczema.” Figure 2 shows that “Very early onset—persistent” eczema displayed 10.4 times higher odds for developing *likelyFA*, followed by “Early onset—persistent” eczema with 3.9 times higher odds (Figure 2). Additionally, “Very early onset—persistent,” “Early onset—remitting” or “Early onset—persistent” eczema phenotypes showed a significantly higher risk of *indeterminateFA*. The results of both “no information on persistence” groups (‘Very early onset—no info’ *N* = 23; ‘Early onset—no info’ *N* = 22) can be found in Supplementary Table S3, together with comprehensive results for all eczema phenotypes.

#### 3.2.1 Association between eczema phenotypes and the presence of asthma and hay fever

**FIGURE 3:**
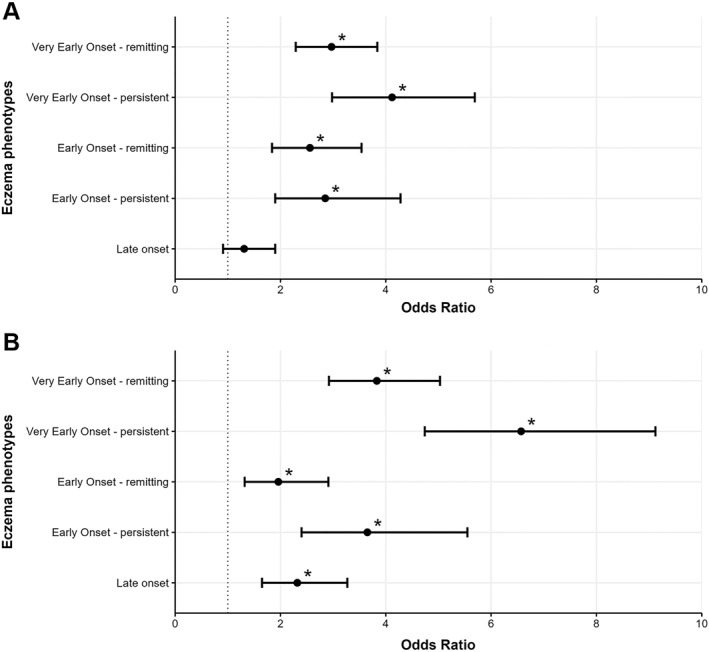
Association of eczema phenotypes with asthma (A) and hay fever (B) presence. Adjusted Odds Ratio (with 95% CI) from the logistic regression analysis are depicted, adjusted for age at baseline, sex, smoking during pregnancy, passive smoking during pregnancy, location of residency in the first 6 months, exposure to a furry/hairy pet in the first 6 months, breast feeding, duration of breastfeeding, asthma presence among the parents, parent's income level, parent's education level. Eczema phenotypes were compared to the “Never eczema” reference group. *N* = 6,638, * *p*‐value <0.05.

Ever eczema was associated with the presence of asthma and hay fever (Supplementary Table S2). All eczema phenotypes besides the “Late onset” group showed significantly higher risks of having asthma (Supplementary Table S4). The ‘Very early onset—persistent’ group with 4.1‐times higher odds observed the highest risk for having asthma (Figure 3A). All eczema phenotypes showed significant associations with hay fever presence (Supplementary Table S5). The “Very early onset—persistent” group observed the highest risk of having hay fever with 6.6‐times higher odds (Figure 3B).

### 3.3 Allergic disease trajectories

**FIGURE 4:**
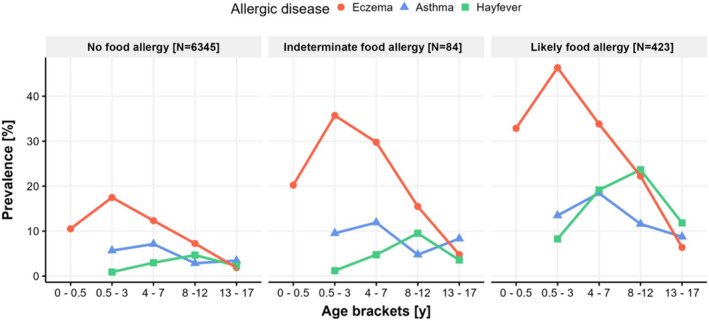
Allergic trajectories of eczema, asthma and hay fever stratified by the presence of food allergy (FA). The prevalence of allergic diseases within each FA presence group was computed and plotted over the age brackets. For asthma and hay fever, there was no data available for the first age bracket, covering the first 6 months of life. *N* = 6,852; y, years.

The reported prevalence of eczema, asthma and hay fever within each age bracket, stratified by FA presence, are presented in Figure 4 and Supplementary Table S6. In all FA groups, the peak in prevalence of eczema was observed in the age bracket 6 months–3 years, followed by a steady decline over the later age brackets. Regarding asthma, the highest prevalence over all groups was observed in the age bracket 4–7 years and for hay fever in the age bracket 8–12 years. Generally, the prevalence of eczema was higher than the prevalence of asthma and hay fever, at least until the age bracket of 4–7 years. Comparing eczema prevalence between the FA groups at each age bracket showed significant differences between the *noFA* and *likelyFA* groups over all age brackets. When comparing the eczema prevalence of the *noFA* group to *indeterminateFA*, only the last age bracket did not show a significant difference. The asthma prevalence showed significant differences across all age brackets, when comparing *noFA* and *likelyFA*, but no significant differences were observed between *noFA* and *indeterminateFA*. The same was seen for hay fever, with significant differences between *noFA* and *likelyFA*, but not between *noFA* and *indeterminateFA*.

## 4 DISCUSSION

### 4.1 Primary findings

In one of the largest population‐based cohort studies on this topic to date, including 6852 children/adolescents, we identified an association of eczema and eczema phenotypes with the presence of FA, asthma and hay fever. “Very early onset—persistent” eczema showed the strongest associations across all investigated allergic diseases. Thus, children who develop eczema before the age of 6 months, which is persistent, are at the highest risk for developing allergic multimorbidity. Moreover, participants with FA observed a significantly higher prevalence of eczema, asthma and hay fever throughout childhood, comparing them to participants without FA.

### 4.2 Interpretation

We confirmed that eczema presence represents a risk factor for FA, as shown previously.^14^, ^15^, ^16^, ^17^ Further, we demonstrated associations eczema presence and presence of asthma and hay fever, reflecting findings from other studies.^14^, ^18^, ^19^ Associations between eczema phenotypes and FA and asthma and hay fever presence revealed, that all phenotypes, especially the persistent ones, had significantly higher risks of developing subsequent allergic disease when compared to children who never had eczema. Other studies also reported that persistent eczema was associated with the development of FA^20^, ^21^ as well as asthma and hay fever.^8^, ^11^, ^21^, ^22^, ^23^ Furthermore, our results suggest that the age of eczema onset plays an important role in allergic multimorbidity. Earlier onset of eczema showed a stronger association with FA, asthma and hay fever presence, with “Very early onset” showing higher aOR's (adjusted Odds Ratio) than “Early onset” and “Late onset”. These findings agree with similar reports, showing eczema onset before 2 years of age to be associated with development of FA, asthma and hay fever.^22^, ^24^, ^25^ Onset of eczema in the first 2 months has even been reported to have the strongest association with development of FA by age 3.^26^

Taken together, our findings suggest that individuals with eczema onset before 6 months and persistent eczema are at the highest risk for developing‐allergic multimorbidity during childhood and adolescence. This supports our hypothesis that this subpopulation might be at the highest risk of sensitization against food or inhalant allergens before allergen tolerance develops. The higher likelihood of allergen sensitization and subsequent development of FA, asthma and hay fever in patients with eczema can be, at least partially, attributed to impairment of the skin's barrier function.^27^, ^28^ The dual allergen exposure hypothesis suggests that, regarding FA, the oral consumption of allergenic foods promotes immune tolerance, whereas exposure to food allergens on the skin (before oral tolerance could develop), is more likely to lead to epicutaneous sensitization through allergen penetration and cytokine dysregulation. This applies especially, but not exclusively, to eczematous skin.^4^, ^27^, ^29^ For asthma and hay fever, evidence regarding epicutaneous sensitization with inhalant allergens is still limited.^6^, ^30^ However, it has been reported that the likelihood of inhalant allergen sensitization is higher in children with eczema because of increased skin permeability, even when skin barrier parameters were measured on non‐lesional skin. The same study described a correlation between allergy score (cumulative skin‐prick‐test results) and eczema duration.^31^ The dual allergen exposure hypothesis has gained traction throughout the last decade and resulted in a change in management from allergen avoidance strategies toward early dietary introduction of allergenic foods, aiming for tolerance induction.^32^ It is important to note, that the clinical manifestation of eczema is not a driving factor for the development of FA, but rather represents a symptom indicating an underlying epithelial barrier dysfunction.^33^ Thus, in theory, restoring barrier function represents a promising preventative strategy for allergic multimorbidity in children with eczema.^34^, ^35^ Thus far, studies evaluating specific interventions in infancy, targeted at preventing‐allergic diseases, have not produced recommendable strategies yet.^36^, ^37^ The use of emollients to strengthen the skin barrier might even lead to a higher risk for FA and skin infections.^36^ Administration of topical corticosteroids to both lesional and non‐lesional skin, in contrast to exclusively treating lesional skin (reactive therapy), was associated with a 10% lower risk of hen's egg allergy at 28 weeks of age. However, this enhanced therapy raised safety concerns regarding physical development, with lowered body weight and height compared to the reactive therapy.^38^ New possibilities for the prevention of subsequent allergic disease in children with eczema are currently being investigated, for example, in the Stopping Eczema and Allergy study (NCT03742414).

However, those who experience the longest duration of skin barrier impairment, such as our “Very early onset—persistent” phenotype, may benefit the most from early eczema prevention and treatment. It would be crucial to find a method or biomarker to identify this subpopulation as early as possible.

The atopic trajectories for eczema, asthma and hay fever, presented in our cohort, closely resemble the typical trajectory of the atopic march across all FA presence groups.^5^ Early peaks of eczema prevalence around the age of 2 years, followed by peaks of asthma prevalence around 5.5 years and peaks of hay fever prevalence around 10 years of age. However, multiple findings have challenged the idea of the strict sequence suggested in the atopic march, which originally stems from epidemiological studies focusing on cumulative trajectories in large cohorts. When profiles of eczema, asthma (wheeze) and hay fever (rhinitis) are analyzed based on individuals, the developmental trajectories display greater heterogeneity.^39^, ^40^ In one study, the identified group closest to the atopic march only covered about 3% of all children enrolled and about 6% of those with any atopic symptoms.^39^ Another study reported early life eczema representing the largest risk factor for allergic multimorbidity, but it only led to multimorbidity in about 25% of cases.^40^ Today, the paradigm of the strictly sequential atopic march is reconsidered and emphasizes that focus should lie on identifying those at highest risk of multimorbidity in early‐life.

### 4.3 Strengths and limitations

This study has some strengths that support the validity of our findings. First, we observed the association of eczema phenotypes with‐allergic multimorbidity in one of the largest cohorts (*N* = 6852) to date. Furthermore, all allergic diseases, which are considered in the atopic march, were included in the analysis. The overall prevalence of allergic diseases that we observed is in line with other reports.^15^, ^16^, ^41^, ^42^, ^43^, ^44^ However, eczema prevalence was slightly higher than commonly reported.^16^, ^18^, ^43^, ^44^ This could be partly due to the self‐/guardian‐reported factors utilized for verification of eczema presence. Furthermore, our investigation covers a wide age‐range (0–17 years) including infancy, childhood, and adolescence, while many other studies assess outcomes at a younger age. Regression models were adjusted comprehensively for confounders, known to influence allergic disease development. Lastly, due to the population‐based study design, our findings are derived from real‐life data and do not rely on selective sampling, leading to results representative for the general population.

However, some limitations need to be addressed. Firstly, the information on the presence of the allergic diseases used in this study was derived from self‐/guardian‐reported questionnaires. For eczema, hay fever, and asthma, information on the presence, medication use and treatment by a doctor was provided. Recall bias may have resulted in over‐ or underestimation of the prevalence. Food allergy presence was defined as previously published by Westerlaken‐van Ginkel^13^ and was categorized into *likelyFA* and *indeterminateFA*. In this classification, specificity was prioritized over sensitivity to reduce the number of false positive cases within the *likelyFA* group.^13^ Additionally, the calculation of age of onset displayed some imprecision, as it was based on the age brackets which had been predefined with differing durations, resulting in estimates within those age brackets. Furthermore, recall bias at baseline may have been present since subjects had to report the presence, treatment and medication of allergic diseases since birth. Lastly, because the Lifelines cohort exclusively consisted of participants based in the Northern Netherlands, our results are subject to a regional bias. However, as described above, our data are in line with findings from comparable cohorts.

### 4.4 Outlook

Future investigations concerned with the effect of early life eczema on allergic multimorbidity should consider the integration of disease severity for eczema, food allergies, asthma and hay fever, since it was repeatedly shown that eczema severity was associated with the development and severity of other allergic diseases.^14^, ^19^, ^24^, ^45^ Further, there seems to be a heterogenic association between eczema phenotypes and different allergenic foods.^25^ This distinction could result in a more detailed understanding of the allergen‐specific associations present among comorbid‐allergic diseases, if applied to future studies.

## 5 CONCLUSION

Our findings show that eczema was significantly associated with FA, asthma and hay fever. The different eczema phenotypes observed varied degrees of association with these allergic diseases and the strongest associations were shown for “Very early onset—persistent” eczema. These results support our hypothesis that children, with very early onset and persistent eczema, are at the highest risk for developing food or inhalant allergies. Ultimately, exploring methods or biomarkers which might aid in the early identification of this subpopulation could increase the efficacy of early prevention and treatment measures, leading to individual based medicine.

## AUTHOR CONTRIBUTIONS

**L. A. Miltner**: Conceptualization; data curation; formal analysis; Investigation; methodology; visualization; writing – original draft; writing – review & editing. **J. M. Vonk**: Conceptualization; data curation; formal analysis; investigation; methodology; software; supervision; validation; visualization; writing – original draft; writing – review & editing. **J. L. van der Velde**: Conceptualization; investigation; methodology; supervision; validation; writing – original draft, writing – review & editing. **A. B. Sprikkelman**: Conceptualization; funding acquisition; investigation; methodology; project administration; supervision; validation; visualization; writing – original draft; writing – review & editing.

## CONFLICT OF INTEREST STATEMENT

AS reports research grants from Nestle Research, Lausanne and Aimmune, outside the submitted work. Her institution received compensation for her consultancy for Sanofi Netherlands and Nestle Research, Lausanne.

## Supporting information

Supporting Information S1
