# Supplementary material for: Eczema in early childhood increases the risk of allergic multimorbidity
Source: Clin Transl Allergy. 2024 Sep 1;14(9):e12384. doi: 10.1002/clt2.12384 (PMC11366446; doi:10.1002/clt2.12384)
Supplement: Supplementary file 1 — Supporting Information S1 [file CLT2-14-e12384-s001.docx]

**Supplement**

**Questionnaire data for evaluating allergic disease presence**

The parameters presence, medication and treatment were assessed for each age bracket using the following questions:

“Did your child suffer from eczema/asthma/hay fever during [age bracket]?”,

“Did your child use any medication for eczema/asthma/hay fever during [age bracket]?” and

“Did your child receive any medical treatment by a doctor for eczema/asthma/hay fever during [age bracket]?”)

**Further details on FA definition**

Classification of allergens:

| **In questionnaire = common allergens** | **Common allergens** | **Less common allergens** |
| --- | --- | --- |
| Wheat | Kiwi | Meat (pork/beef) |
| Cowsmilk | Strawberry | Chocolate/cacao |
| Egg | Cherry | Pineapple |
| Soy (milk) | Pear | Tomato |
| Apple | Peach | Bellpepper |
| Sesame | Fruit (unspecified) | Sugar/fructose |
| Peanut | Banana | MSG/chinese food |
| Almond | Carrot (peal) | Gluten |
| Walnut | Stonefruit/drupes | Melon (all types) |
| Cashewnut | Nectarine | Onion |
| Hazelnut | Mixed nuts/nuts/”studentenhaver” /chocolate with nuts | Herbs/spices/curry(powder)/cinnamon |
| Pistacchio | Brazil nut/Paranut | Lactose |
| Fish (1 or more types) | Pinenut | potato (peel) |
| Shellfish | Papaya | cheese (alle sorts) |
|  | Dairy products (except cheese) | citrusfruits/orange/orangejuice |
|  | Celery | Cabbage |
|  | Pecannut | Mushrooms |
|  | Maize/Corn | Additives (flavoring/coloring/sweeteners etc) |
|  | Macadamianut | Wine/histamine/sulphite (wine_sulphite) |
|  | Plum | Pungent spices/pepper |
|  | Avocado | Coffee/caffeine/tea |
|  | Mango | Fat |
|  | Apricot | Garlic |
|  | Olive | Spinach |
|  | Dade | Yeast |
|  | Blackberry | Alcohol |
|  | Raspberry | Lettuce/aragula |
|  | Gojiberry | Nightshade/aubergine |
|  | Coco(nut) | Rye |
|  | Berries (all types) | Grapes |
|  | Almond paste | Legumes |
|  | Sataysauce | Honey |
|  | Peel of nuts | Beer |
|  | Chiaseed | Ice cream |
|  | Poppyseed | Radish |
|  | Seeds in bread | Rice |
|  | Soy products (Soy-meat substitutes, ketjap, tofu, quorn) | Arachideoil |
|  | Sushi (fish) | Sunfloweroil |
|  | Artificila Crab/surimi (shellfish) | Asparagus |
|  | Kaki fruit/persimmon/starfruit | Coca cola |
|  | Lupine | Gelatin |
|  | Lychee | Kefir (other) |
|  | Passionfruit | Quinine |
|  | Maracuja | Chicken/poultry |
|  | Pumpkinseeds | Combination of (animal) proteins and carbohydrates (other) |
|  | Quinoa (seed) | Cereals (grain) |
|  | Sunflowerseed | Whole grain products |
|  | Exotic fruit | Whitebread |
|  | Pomegranate | Vegetables/brocoli/cauliflower/endive |
|  | Oystersauce | Horsemilk |
|  | Buckwheat | Goatmilk |
|  | Italian pasta |  |
|  | Applejuice |  |
|  | Apple pie |  |
|  | Eggyolk |  |
|  | Fig |  |

Note: only the food mentioned in this variable was scored and any interpretation or additional information given by the participant was not taken into account.

Classification of allergic reactions (and affected organs):

| **In questionnaire  = common reaction** | **Common reaction** | **Less common reaction** |
| --- | --- | --- |
| Dizziness | Painful mouth/tongue/”blisters” in mouth | Headache/migraine (ns) |
| Palpitations | Increase saliva/mucus (without reporting CMA or dairy products allergy) | Puffy feeling/swollen belly |
| Loss of consciousness | Red/Swollen eyes | Tired/drowsy |
| Nausea | Sneezing | Stomach ache/heartburn |
| Abdominal cramps | Strange feeling or painful throat | Painful abdomen/intestines |
| Vomiting | Swollen face/ Quincke’s edema | Pimples/acne/bumps |
| Diarrhea | Swollen throat | Flatulence |
| Itch in mouth, ears, or throat | Smothery | Constipation |
| Itching tongue or lips | Swelling hands/feet | Mouth ulcers |
| Itching or tearing eyes | Red bumps (hives) | Increased saliva/mucus (with reporting CMA or dairy products allergy) |
| Swollen tongue or lips | Itchy palate | Painful joints |
| Feeling of throat closure | Edema/ generalized swelling | Not feeling well/depressed/bad mood |
| Wheeze | Strange feeling in mouth | Change in stool (frequency/composition) |
| Dyspnea | Problems swallowing | Sweating |
| Cough | Change of voice | Feeling restless |
| Nose symptoms | Swelling of tongue/mouth | Painful muscles |
| (increase) eczema on several locations or whole body | Swollen ears | Strange taste in mouth (bitter/chemical/bad) |
| Itchy skin on several locations or whole body | Rash on face, chest and neck | Chest pain/pressure on chest |
| Redness of skin on several locations or whole body | Metallic taste in mouth | Fluid retention |
| Urticaria on several locations or whole body | Anaphylactic shock | Increase in temperature/fever |
|  | Balance problems | Painful/burning/irritated oesophagus |
|  | Hayfever | Burping |
|  | Aphthous ulcer in mouth | Swollen glands/mucous membranes |
|  | Getting pale | Cold sores |
|  | Loss of sense in mouth or lips | Infected sinus |
|  | Colic attack | Dry skin/throat/mouth/eyes |
|  | Eczema/itching/redness without further information (for children/adolescents) | Eczema/itching/redness without further information (for adults) |
|  |  | Swollen/painful teeth/gums |

Note: only the reaction mentioned in this variable was scored and any interpretation or additional information given by the participant was not taken into account.

Participants were assigned to the *indeterminateFA* group if they did not meet the requirements for the likelyFA group. Thus, they had stated:

- only symptoms to foods uncommon or unproven to be elicitors of immediate allergic reactions
- only symptoms other than those consistent with immediate allergic reactions to foods
- only symptoms and/or foods associated with other disorders (e.g. gluten with celiac disease)
- one or more other (diagnostic) characteristics which are not consistent with allergic reactions to food

In contrast to the definition by Westerlaken van Ginkel *et al.* for adults, characteristics assessing FA presence in children and adolescents was limited to two factors: the person diagnosing the FA and the time between ingesting the food in question and onset of symptoms. *LikelyFA* was rejected if the FA was only diagnosed by an alternative practitioner (e.g. homeopathic, chiropractic, osteopathic) and/or the onset of symptoms after ingestion exceeded one day. Participants lacking information on FA presence were excluded from the analysis.

**R Studio packages**

- Hadley Wickham, Evan Miller and Danny Smith (2022). **haven**: Import and Export ‘SPSS’, ‘Stata’ and ‘SAS’ Files R package version 2.5.0. https://CRAN.R-project.org/package=haven
- Wickham et al., (2019). Welcome to the **tidyverse**. Journal of Open Software, 4(43), 1686, https://doi.org/10.21105/joss.01686
- Hadley Wickham, Romain François, Lionel Henry and Kirill Müller (2022). **dplyr**: A Grammar of Data Manipulation. R package version 1.0.9. https://CRAN.R-project.org/package=dplyr
- Gregory R. Wanes, Ben Bolker Thomas Lumley, Randall C. Johnson. Contributions from Randall C. Johnson are copyright SAIC-Frederick, Inc. Funded by the Intramural Research Program, of the NIH, National Center Institute and Center for Cancer Reseach under NCI Contract NO1-CO-12400. (2022). **gmodels**: Various R Programming Tools. https://CRAN.R-project.org/package=gmodels
- Matt Dowle and Arun Srinivasan (2021). **data.table**: Extension of ‘data.frame’. R package version 1.14.2. https://CRAN.R-project.org/package=data.table
- H.Wickham. **ggplot2**: Elegant Graphics for Data Analysis. Springer-Verlag New York, 2016.
- Revelle, W. (2022) **psych**: Procedures for Personality and Psychological Research, Northwestern Universit, Evanston, Illinois, USA, https://CRAN.R-project.org/package=psych
- Hadley Wickham (2007). Reshaping Data with the **reshape** Package. Journal of Statistical Software, 21(12), 1-20. URL https://www.jstatsoft.org/v21/i12/.
- Phillip Schauberger and Alexander Walker (2021). **openxlsx**: Read, Write and Edit xlsx Files. R package version 4.2.5. https://CRAN.R-project.org/package=openxlsx
- Jeroen Ooms (2021). **writexl**: Export Data Frames to Excel ‘xlsx’ Format. R package version 1.4.0. https://CRAN.R-project.org/package=writexl
- Venables, W. N. & Ripley, B. D. (2002), (**nnet**) Modern Applied Statistics with S. Fourth Edition. Springer, New York. ISBN 0-387-95457-0
- Max Kuhn (2022). **caret**: Classification and Regression Training. R package version 6.0-92. https://CRAN.R-project.org/package=caret
- Thomas Lin Pedersen (2021). **ggforce**: Accelerating ‘ggplot2’. R package version 0.3.3. https://CRAN.R-project.org/package=ggforce
- Alboukadel Kassambara (2021). **rstatix**: Pipe-Friendly Framework for Basic Statistical Tests. R package version 0.7.0. https://CRAN.R-project.org/package=rstatix
- Venables, W. N. & Ripley B. D. (2002), (**MASS**) Modern Applied Statistics with S. Fourth Edition. Springer, New York. ISBN 0-387-95457-0
- Claus O. Wilke (2020). **ggtext**: Improved Text Rendering Support for ‘ggplot’. R package version 0.1.1. https://CRAN.R-project.org/package=ggtext

**Figures**


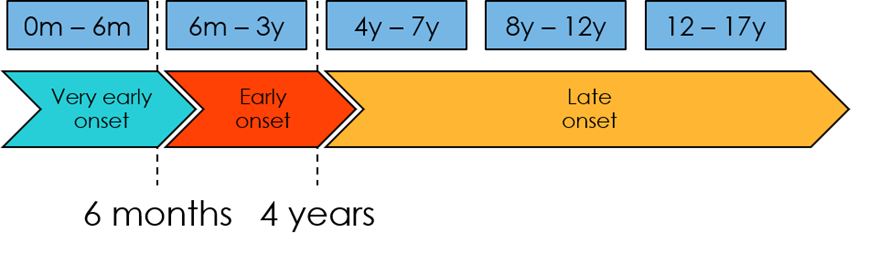


Supplementary Figure 1 Definition of the different groups for the time of eczema onset. m = months, y = years.


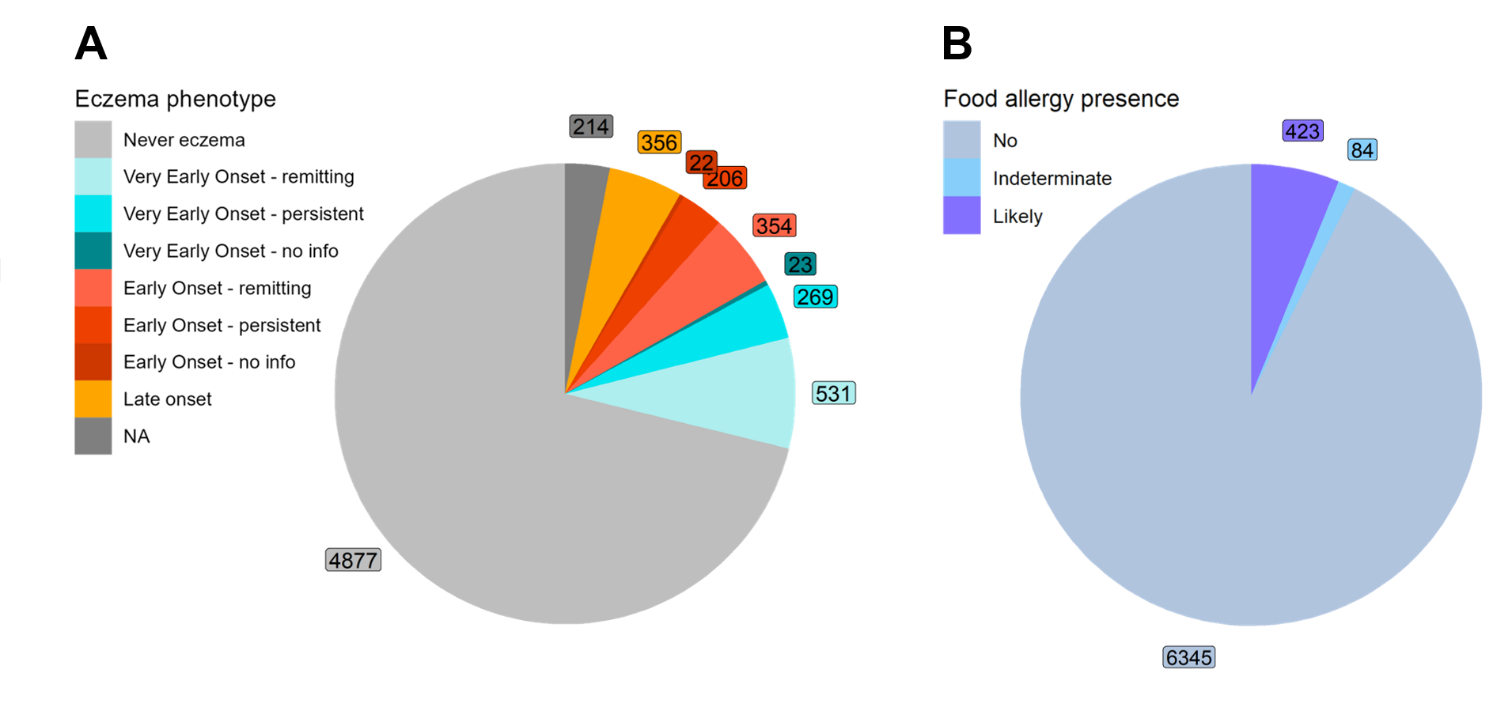


Supplementary Figure 2 Distribution of eczema phenotypes (A) and FA presence (B) in the study population. N = 6852.

**Tables**

Supplementary Table 1 Age brackets included in the different questionnaire versions.

| Questionnaire version | Age brackets covered |
| --- | --- |
| 0 | 0 – 6 m |
| 1 | 6 m – 3 y, 4 y – ageQ |
| 2 | 6 m – 3 y, 4 – 7 y, 8 y – ageQ |
| 3 | 6 m – 3 y, 4 – 7 y, 8 – 12 y, 13 y – ageQ |

ageQ = age at time of questionnaire; m = months; y = years.

Supplementary Table 2 Results from the multinomial regression (indeterminate FA and likely FA) and logistic regression (asthma and hay fever) analysis testing the associations between eczema presence and presence of other allergic diseases.

| Eczema | Allergic disease |  | OR (95% CI) | p-value |  | aOR (95% CI) | adj. p-value |
| --- | --- | --- | --- | --- | --- | --- | --- |
| yes | Indeterminate FA |  | 2.26 (1.46 – 3.49) | < 0.001 |  | 2.35 (1.50 – 3.67) | < 0.001 |
|  | Likely FA |  | 3.72 (3.05 – 4.55) | < 0.001 |  | 3.74 (3.04 – 4.60) | < 0.001 |
|  | Asthma |  | 2.61 (2.22 – 3.06) | < 0.001 |  | 2.60 (2.19 – 3.09) | < 0.001 |
|  | Hay fever |  | 3.30 (2.77 – 3.94) | < 0.001 |  | 3.33 (2.77 – 4.01) | < 0.001 |

Adjusted for age at follow-up (FA)/ age at baseline (asthma, hay fever), sex, location in the first 6 months, exposure to fury hairy pets in the first 6 months, breastfeeding, breastfeeding duration, pregnant smoking, pregnant passive smoking, parents’ asthma, parents’ income, parents’ education. FA = food allergy, OR = Odds Ratio, aOR = adjusted Odds Ratio, CI = confidence interval.

Supplementary Table 3 Results of the multinomial regression analysis testing the association between eczema phenotypes and FA presence.

| Eczema Phenotype | FA presence |  | OR (95% CI) | p-value |  | aOR (95% CI) | adj. p-value |
| --- | --- | --- | --- | --- | --- | --- | --- |
| Very early onset - remitting | indeterminate |  | 2.03 (0.99 – 4.18) | 0.055 |  | 2.08 (0.97 – 4.48) | 0.061 |
|  | likely |  | 3.51 (2.6 – 4.74) | < 0.001 |  | 3.71 (2.72 – 5.05) | < 0.001 |
| Very early onset - persistent | indeterminate |  | 4.39 (2.04 – 9.45) | < 0.001 |  | 5.37 (2.45 – 11.75) | < 0.001 |
|  | likely |  | 9.75 (7.17 – 13.27) | < 0.001 |  | 10.40 (7.52 – 14.38) | < 0.001 |
| Very early onset - no info | indeterminate |  | NA† |  |  | NA † |  |
|  | likely |  | 2.39 (0.56 – 10.28) | 0.241 |  | 2.76 (0.63 – 12.05) | 0.177 |
| Early onset - remitting | indeterminate |  | 2.3 (1.03 – 5.15) | 0.042 |  | 2.60 (1.15 – 5.86) | 0.022 |
|  | likely |  | 2.64 (1.79 – 3.89) | < 0.001 |  | 2.86 (1.93 – 4.24) | < 0.001 |
| Early onset - persistent | indeterminate |  | 4.83 (2.24 – 10.41) | < 0.001 |  | 5.39 (2.46 – 11.81) | < 0.001 |
|  | likely |  | 3.97 (2.58 – 6.11) | < 0.001 |  | 3.91 (2.49 – 6.15) | < 0.001 |
| Early onset – no info | indeterminate |  | NA † |  |  | NA † |  |
|  | likely |  | 1.2 (0.16 – 8.94) | 0.861 |  | 1.25 (0.16 – 9.46) | 0.831 |
| Late onset | indeterminate |  | 1.26 (0.45 – 3.52) | 0.66 |  | 1.17 (0.41 – 3.30) | 0.767 |
|  | likely |  | 1.84 (1.18 – 2.85) | 0.007 |  | 1.63 (1.04 – 2.57) | 0.035 |

Adjusted for age at follow-up, sex, location in the first 6 months, exposure to fury hairy pets in the first 6 months, breastfeeding, breastfeeding duration, pregnant smoking, pregnant passive smoking, parents’ asthma, parents’ income, parents’ education. † not enough cases to reliably estimate this OR. OR = Odds Ratio, aOR = adjusted Odds Ratio, CI = confidence interval.

Supplementary Table 4 Results of the logistic regression analysis testing the association between eczema phenotypes and asthma presence.

| Eczema Phenotype |  | OR (95% CI) | p-value |  | aOR (95% CI) | adj. p-value |
| --- | --- | --- | --- | --- | --- | --- |
| Never eczema |  | reference |  |  | reference |  |
| Very early onset - remitting |  | 2.98 (2.34 – 3.8) | < 0.001 |  | 2.97 (2.29 – 3.84) | < 0.001 |
| Very early onset - persistent |  | 4.05 (3 – 5.46) | < 0.001 |  | 4.12 (2.98 – 5.69) | < 0.001 |
| Very early onset - no info |  | 3.53 (1.3 – 9.65) | 0.013 |  | 4.01 (1.38 – 11.62) | 0.011 |
| Early onset - remitting |  | 2.34 (1.72 – 3.18) | < 0.001 |  | 2.56 (1.84 – 3.54) | < 0.001 |
| Early onset - persistent |  | 2.51 (1.71 – 3.68) | < 0.001 |  | 2.85 (1.9 – 4.28) | < 0.001 |
| Early onset – no info |  | 1.27 (0.3 – 5.45) | 0.748 |  | 1.87 (0.42 – 8.2) | 0.409 |
| Late onset |  | 1.47 (1.03 – 2.11) | 0.034 |  | 1.31 (0.91 – 1.9) | 0.145 |

Adjusted for age at baseline (asthma, hay fever), sex, location in the first 6 months, exposure to fury hairy pets in the first 6 months, breastfeeding, breastfeeding duration, pregnant smoking, pregnant passive smoking, parents’ asthma, parents’ income, parents’ education. OR = Odds Ratio, aOR = adjusted Odds Ratio, CI = confidence interval.

Supplementary Table 5 Results of the logistic regression analysis testing the association between eczema phenotypes and hay fever presence.

| Eczema Phenotype |  | OR (95% CI) | p-value |  | aOR (95% CI) | adj. p-value |
| --- | --- | --- | --- | --- | --- | --- |
| Never eczema |  | reference |  |  | reference |  |
| Very early onset - remitting |  | 3.64 (2.82 – 4.71) | < 0.001 |  | 3.83 (2.92 – 5.03) | < 0.001 |
| Very early onset - persistent |  | 5.96 (4.41 – 8.06) | < 0.001 |  | 6.57 (4.74 – 9.12) | < 0.001 |
| Very early onset - no info |  | 0.8 (0.11 – 5.96) | 0.828 |  | 0.92 (0.12 – 7.08) | 0.933 |
| Early onset - remitting |  | 1.87 (1.29 – 2.72) | 0.001 |  | 1.96 (1.32 – 2.91) | 0.009 |
| Early onset - persistent |  | 3.36 (2.27 – 4.98) | < 0.001 |  | 3.65 (2.40 – 5.55) | < 0.001 |
| Early onset – no info |  | 1.76 (0.41 – 7.58) | 0.447 |  | 2.34 (0.53 – 10.44) | 0.264 |
| Late onset |  | 2.68 (1.92 – 3.73) | < 0.001 |  | 2.32 (1.65 – 3.27) | < 0.001 |

Adjusted for age at baseline (asthma, hay fever), sex, location in the first 6 months, exposure to fury hairy pets in the first 6 months, breastfeeding, breastfeeding duration, pregnant smoking, pregnant passive smoking, parents’ asthma, parents’ income, parents’ education. OR = Odds Ratio, aOR = adjusted Odds Ratio, CI = confidence interval.

Supplementary Table 6 Prevalence of eczema, asthma and hay fever for each age bracket stratified by the FA presence groups.

| Age bracket [y] |  | Prevalence [%] | | | | | | | | | | | | | | | |  |
| --- | --- | --- | --- | --- | --- | --- | --- | --- | --- | --- | --- | --- | --- | --- | --- | --- | --- | --- |
|  | **Eczema** | | | | | | **Asthma** | | | | | **Hay fever** | | | | | | |
|  |  | **No FA** | **Ind. FA** | **p-value**  **ind. FA** | **Likely FA** | **p-value**  **likely FA** | **No FA** | **Ind. FA** | **p-value**  **ind. FA** | **Likely FA** | **p-value**  **likely FA** | | **No FA** | **Ind. FA** | **p-value**  **ind. FA** | **Likely FA** | **p-value**  **likely FA** |  |
| 0 – 0.5 |  | 10.5 | 20.2 | 0.012 | 32.9 | < 0.001 | NA | NA | NA | NA | NA | | NA | NA | NA | NA | NA |  |
| 0.5 – 3 |  | 17.5 | 35.7 | < 0.001 | 46.3 | < 0.001 | 5.7 | N<10 | 0.415 | 13.5 | < 0.001 | | 0.9 | N<10 | 1.000 | 8.3 | < 0.001 |  |
| 4 – 7 |  | 12.3 | 29.8 | < 0.001 | 33.8 | < 0.001 | 7.1 | 11.9 | 0.289 | 18.4 | < 0.001 | | 3.0 | N<10 | 0.532 | 19.1 | < 0.001 |  |
| 8 – 12 |  | 7.3 | 15.5 | 0.039 | 22.2 | < 0.001 | 2.9 | N<10 | 0.587 | 11.6 | < 0.001 | | 4.7 | N<10 | 0.112 | 23.6 | < 0.001 |  |
| 13 – 17 |  | 1.9 | N<10 | 0.498 | 6.4 | < 0.001 | 3.5 | N<10 | 0.111 | 8.8 | < 0.001 | | 2.3 | N<10 | 0.621 | 11.8 | < 0.001 |  |

N<10: Due to privacy protection, categories with less than 10 subjects are displayed as N<10. NA = not available, y = years, FA = food allergy, Ind.FA = indeterminate FA.
